# Deep learning-based automatic detection of pancreatic ductal adenocarcinoma ≤ 2 cm with high-resolution computed tomography: impact of the combination of tumor mass detection and indirect indicator evaluation

**DOI:** 10.1007/s11604-025-01836-z

**Published:** 2025-07-18

**Authors:** Mizuki Ozawa, Miyuki Sone, Susumu Hijioka, Hidenobu Hara, Yusuke Wakatsuki, Toshihiro Ishihara, Chihiro Hattori, Ryo Hirano, Shintaro Ambo, Minoru Esaki, Masahiko Kusumoto, Yoshiyuki Matsui

**Affiliations:** 1 Department of Diagnostic Radiology, National Cancer Center Hospital, 5-1-1, Tsukiji, Chuo-ku, Tokyo 1040045 Japan; 2 Cancer Medicine, Jikei University Graduate School of Medicine, Tokyo, Japan; 3 Department of Hepatobiliary and Pancreatic Oncology, National Cancer Center Hospital, 5-1-1, Tsukiji, Chuo-ku, Tokyo 1040045 Japan; 4 Department of Gastroenterology, Yokohama City Minato Red Cross Hospital, Yokohama, Japan; 5 Department of Diagnostic Technology, National Cancer Center Hospital, 5-1-1, Tsukiji, Chuo-ku, Tokyo 1040045 Japan; 6 Canon Medical Systems Corporation, Tochigi, Japan; 7 Department of Hepatobiliary and Pancreatic Surgery, National Cancer Center Hospital, 5-1-1, Tsukiji, Chuo-ku, Tokyo 1040045 Japan; 8 Department of Urology, National Cancer Center Hospital, 5-1-1, Tsukiji, Chuo-ku, Tokyo 1040045 Japan

**Keywords:** Pancreatic ductal adenocarcinomas, Computed tomography, Tumor mass detection, Deep learning

## Abstract

**Purpose:**

Detecting small pancreatic ductal adenocarcinomas (PDAC) is challenging owing to their difficulty in being identified as distinct tumor masses. This study assesses the diagnostic performance of a three-dimensional convolutional neural network for the automatic detection of small PDAC using both automatic tumor mass detection and indirect indicator evaluation.

**Materials and methods:**

High-resolution contrast-enhanced computed tomography (CT) scans from 181 patients diagnosed with PDAC (diameter ≤ 2 cm) between January 2018 and December 2023 were analyzed. The D/P ratio, which is the cross-sectional area of the MPD to that of the pancreatic parenchyma, was identified as an indirect indicator. A total of 204 patient data sets including 104 normal controls were analyzed for automatic tumor mass detection and D/P ratio evaluation. The sensitivity, specificity, positive predictive value (PPV), and negative predictive value (NPV) were evaluated to detect tumor mass. The sensitivity of PDAC detection was compared with that of the software and radiologists, and tumor localization accuracy was validated against endoscopic ultrasonography (EUS) findings.

**Results:**

The sensitivity, specificity, PPV, and NPV for tumor mass detection were 77.0%, 76.0%, 75.5%, and 77.5%, respectively; for D/P ratio detection, 87.0%, 94.2%, 93.5%, and 88.3%, respectively; and for combined tumor mass and D/P ratio detections, 96.0%, 70.2%, 75.6%, and 94.8%, respectively. No significant difference was observed between the software’s sensitivity and that of the radiologist’s report (software, 96.0%; radiologist, 96.0%; *p* = 1). The concordance rate between software findings and EUS was 96.0%.

**Conclusions:**

Combining indirect indicator evaluation with tumor mass detection may improve small PDAC detection accuracy.

**Supplementary Information:**

The online version contains supplementary material available at 10.1007/s11604-025-01836-z.

## Introduction

Despite advancements in diagnostics and therapeutics, pancreatic ductal adenocarcinoma (PDAC) remains one of the most lethal malignancies, with a dismal 5-year survival rate of only 6% [1]. The Union for International Cancer Control 8th edition (UICC), classifies tumors smaller than 2 cm as T1 stage and are commonly recognized as small PDAC [2]. The 5-year survival rate for small PDAC is significantly higher at 30–60% [2], thereby suggesting that the detection of small PDAC may further increase survival rates.

Computed tomography (CT) plays a critical role in the diagnosis of PDAC. However, the CT characteristics of small PDAC are often subtle, which can make diagnosis sometimes difficult even for experienced radiologists [3]. Studies indicate that up to 40% of small PDACs are missed on CT [4], underscoring the difficulties associated with their detection. Given the challenges of small PDAC detection, artificial intelligence, particularly deep learning, has been explored as a potential solution. Although studies have reported good diagnostic performance for automated detection methods [5–9], their sensitivity in detecting small PDAC remains suboptimal ranging from 74.7 to 96.0% [5–7, 9]. Furthermore, these methods are not widely implemented in daily clinical practices.

Small PDACs are often challenging to detect as distinct tumor masses. Consequently, indirect indicators, such as dilatation of the main pancreatic duct (MPD) and atrophy of the pancreatic parenchyma, may play a crucial in diagnosis [10, 11]. For example, compared with the detection rates of 51.5% for tumor masses of small PDAC, that for MPD dilatation is relatively high at 79.6% [10]. Thus, we hypothesized that combining indirect indicators and automatic tumor mass detection could improve the diagnostic accuracy of small PDAC. Furthermore, high-resolution CT (HR-CT), known for its superior low-contrast detectability, may further improve the diagnosis of small PDAC.

This study investigated the diagnostic performance of a three-dimensional convolutional neural network (3D-CNN) applied to HR-CT for the automatic detection of small PDAC while incorporating both tumor mass detection and indirect indicator evaluation.

## Materials and methods

This study was approved by the institutional review board of the author’s affiliated institution (approval number: NCCH-2023-407). The requirement for informed consent was waived because of the retrospective nature of the study.

### Overview of this study

**Fig. 1:**
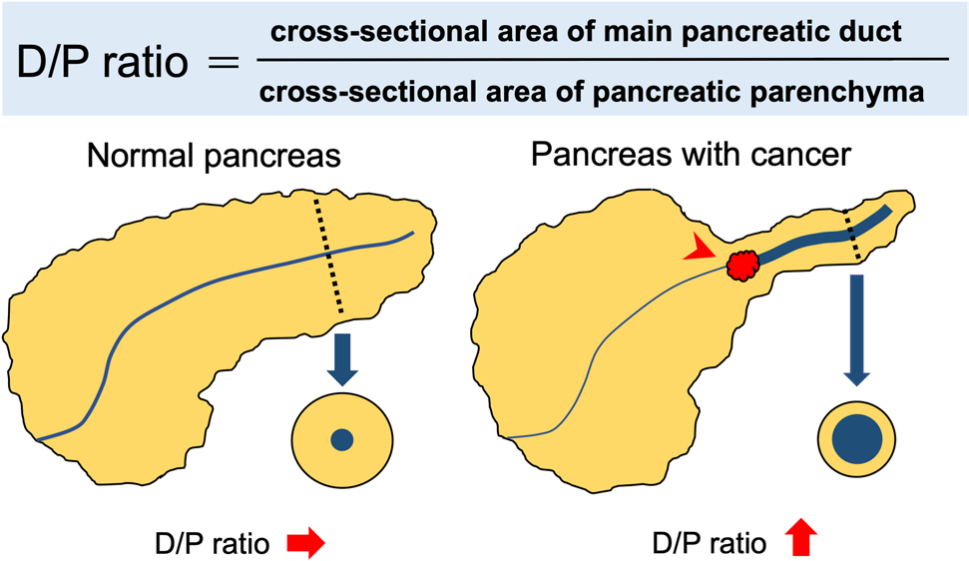
Schematic image of D/P ratio. The D/P ratio is the ratio of the cross-sectional area of the main pancreatic duct (MPD) to the pancreatic parenchyma. The MPD is dilated due to obstruction of the tumor (red arrowhead) and atrophy of the parenchyma due to inflammation. Consequently, the D/P ratio is elevated in pancreatic ductal adenocarcinoma

This study employed a combination of two techniques to improve the detectability of small PDAC. The first approach involved automatic PDAC detection utilizing a three-dimensional convolutional neural network (3D-CNN) based on residual squeeze and excitation U-Net (residual SE U-Net) to identify potential tumor regions. A heat map was generated as a voxelwise likelihood map to indicate the probable PDAC region. In this study, the maximum value of the heatmap was utilized to evaluate the detectability of small PDAC considering the characteristics of the output of CNN model. The detailed CNN architecture and hyperparameters are described in Figure S1 and Table S1 in the supplementary file. The second technique involved evaluating the ratio of the cross-sectional area of the MPD to that of the pancreatic parenchyma (D/P ratio). The D/P ratio is a novel indirect indicator of PDAC that leverages the automated MPD segmentation technology developed in this study [12]. Typically, PDAC is associated with MPD in dilatation and pancreatic parenchyma atrophy. In this study, the D/P ratio was found to be higher in patients with PDAC (Fig. 1). The D/P ratio was calculated in three steps. The first step involved the segmentation of pancreatic parenchyma and MPD. The second step focused on estimating the centerline of the pancreas based on the segmented pancreatic and MPD regions. Finally, the third step entailed calculating the D/P ratio. The D/P ratio was calculated for all cross sections perpendicular to the identified centerline. In this study, the 90th percentile value of the D/P ratios across all cross sections was utilized to evaluate the detectability of small PDAC. The detailed algorithm for calculating the D/P ratio has been described elsewhere [13]. A logical disjunction was used to determine whether PDAC or not, a patient is considered positive if they have a maximum heat map value above the threshold or the D/P ratio above the 90th percentile threshold.

### Patient data set

**Fig. 2:**
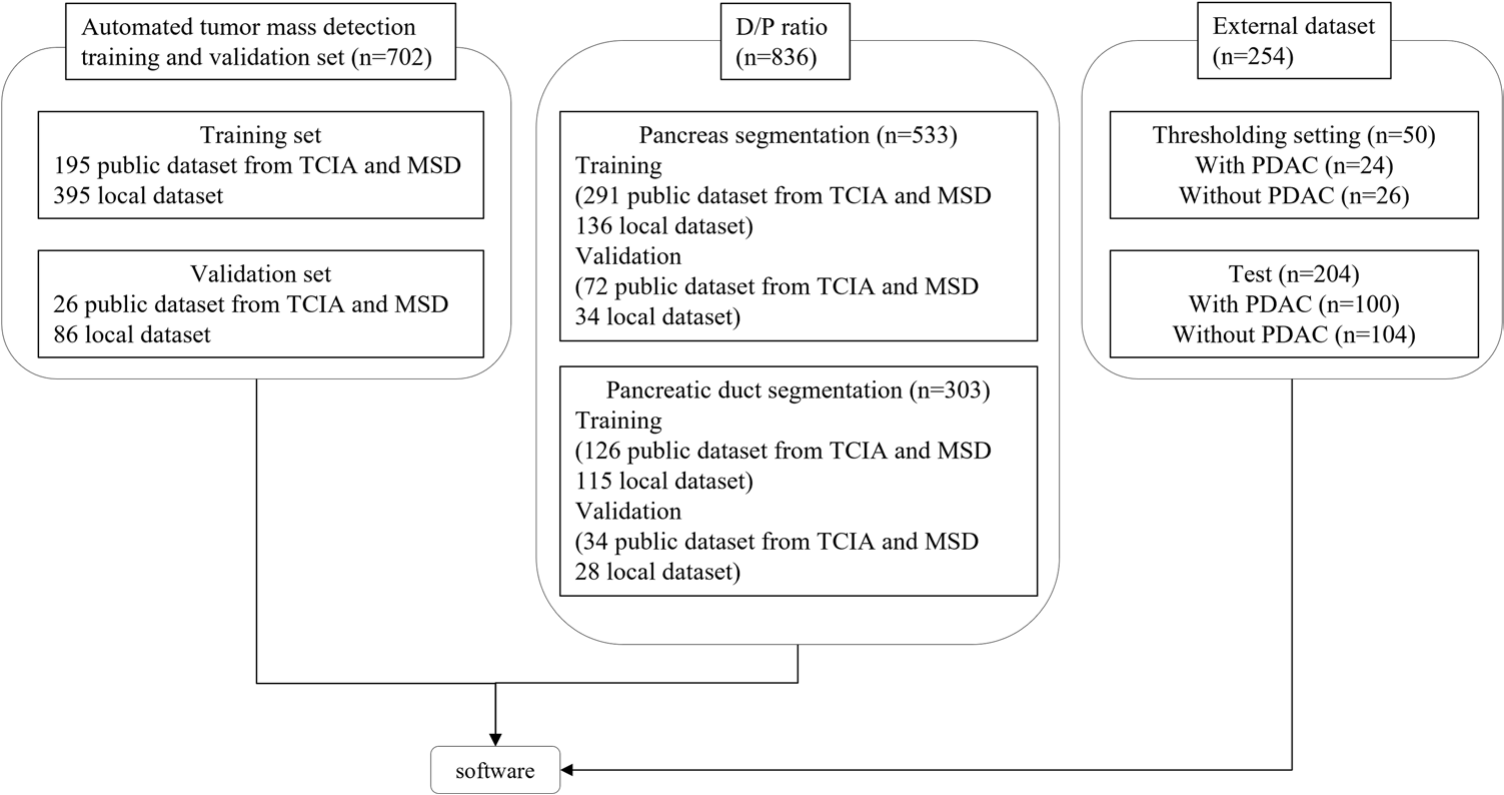
Overview of the study design and patient data set. *TCIA* the cancer imaging archive, *MSD* medical segmentation decathlon, *PDAC* pancreatic ductal adenocarcinoma

Characteristics of the data set that was used to develop the software are summarized in Fig. 2. Contrast-enhanced pancreatic protocol HR-CT scans from 181 consecutive patients diagnosed with T1 stage PDAC by endoscopic ultrasonography (EUS) between January 2018 and December 2023 at our institution were identified as an external data set. Of these, 100 patients were included in this study. The inclusion criteria were as follows: (a) a maximum PDAC diameter of ≤ 2 cm on axial image of CT and (b) age ≥ 18 years. The exclusion criteria were as follows: (a) a clinical history of pancreatic surgery or chemotherapy for PDAC and (b) inappropriate CT images phase (lack of pancreatic phase) or thickness. A total of 104 consecutive patients without pancreatic abnormalities were selected as controls. Additionally, 24 patients with PDAC and 26 patients without pancreatic abnormalities, who were not included in this study, were used to determine the thresholds for both tumor mass detection and the D/P ratio. The same imaging protocols were applied to these 50 patients for threshold setting as the patients for evaluation. All cases of PDAC were pathologically proven, and all cases without PDAC were determined by the consensus between two radiologists (M.O. and M.S., with 11 and 34 years of experience, respectively). Prior to the external test, data from 1538 patients were extracted for the training and validation sets from the following public data sets: The Cancer Imaging Archive (TCIA) and Medical Segmentation Decathlon (MSD) [14, 15].

### CT image acquisition and image reconstruction

In this study, the term “HR-CT” refers to dynamic contrast-enhanced CT with thin-slice (≤ 1 mm) reconstruction performed on various modern multidetector CT systems. While ultra-high-resolution CT using the Aquilion Precision was performed in a subset of cases, we adopted “HR-CT” as a collective term for all scans meeting the high spatial resolution criteria, to maintain consistency and avoid overgeneralization.

Seventy-eight CT images (78%, 78/100) were acquired using the ultra-high or high-resolution mode of a high-spatial-resolution CT (HSR-CT) scanner (Aquilion Precision; Canon Medical Systems, Otawara, Japan). The parameters employed were as follows: 1792 channels, 0.25 mm × 160 rows detector; slice thickness 1 mm; pitch factor of 0.569 or 0.813; gantry rotation period of 0.5 s; matrix size of 1024 × 1024 pixels; X-ray voltage of 120 kV; tube current optimized with automatic exposure control (Volume EC, Canon Medical Systems) using a noise level (standard deviation [SD]) of 12-HU with a thickness of 5.0 mm and a maximum value of 310 mA; and an arbitrary field of view. Some CT images were acquired using a different CT scanner (Aquilion ONE (5%, 5/100); Aquilion PRIME (17%, 17/100); Canon Medical Systems). The parameters employed were as follows: 896 channels; 0.5 mm × 80 rows detector; slice thickness of 1 mm; pitch factor of 0.813; gantry rotation period of 0.5 s; matrix size of 512 × 512 pixels; X-ray voltage 120 kVp; tube current optimized with automatic exposure control (Volume EC, Canon Medical Systems) using a noise level (Standard Deviation) of 12-HU with a thickness of 5.0 mm and a maximum value of 310 mA; and an arbitrary field of view.

The contrast-enhanced CT images were obtained using the following protocol. The first scan was initiated with the bolus-tracking method when the region of interest in the aorta achieved 150-HU. Subsequent scans were performed 20 and 45 s after the first scan, corresponding to the pancreatic and portal venous phases, respectively. The final scan was performed 180 s after the administration of the contrast agent. The contrast material, consisting of 600 mg of iodine per kilogram of total body weight, was injected over 30 s using a 20- or 22-gauge needle and a power injector (Dual shot; Nemoto Kyorindo, Tokyo, Japan). CT images were reconstructed using either hybrid iterative reconstruction (Adaptive Iterative Dose Reduction Three-Dimensional (3D) [AIDR3D, standard setting]; Canon Medical Systems) or deep learning reconstruction (Advanced Intelligent Clear-IQ Engine [AiCE, body sharp, mild]; Canon Medical Systems).

### Evaluation and statistical analysis

Pancreatic phase of CT images was used for CNN input and the D/P ratio calculation. For tumor mass detection, each case was judged as pancreatic cancers if the maximum value of the heat map exceeded the threshold, and as normal otherwise. For the D/P ratio, each case was judged as pancreatic cancers if the 90th percentile value exceeded the threshold, and as normal otherwise. The threshold values were set so that the sensitivity for tumor mass detection was 80% and the specificity for the D/P ratio was 100% in threshold-setting cases. This is because the target of the heat map was to detect pancreatic cancers with high sensitivity, and the target of the D/P ratio was to detect pancreatic cancers that would keep the specificity of the heatmap alone when judged in combination with the heat map. The sensitivity, specificity, positive predictive value (PPV), and negative predictive value (NPV) with 95% confidence interval (CI) were calculated to evaluate the performance of each model: tumor mass detection, D/P ratio, and the combination of tumor mass detection and D/P ratio. The exact McNemar test was used to compare the sensitivity of the software with that of the radiologists as reported in the original radiology report for the same CT studies [6]. All radiology reports were retrieved from electronic health records and were interpreted by board-certified radiologists at a tertiary referral center with extensive experience in managing PDAC cases. Receiver operating characteristic (ROC) curve analyses were also performed. In addition, sensitivity, specificity, PPV, and NPV with 95% CI were calculated for each CT scanner, reconstruction technique, and tumor location. Each tumor location was classified into four patterns: head (uncinate process), head (the other), body, and tail using the CT images. Fisher’s exact test was used to compare the sensitivity, specificity, PPV, and NPV between classified groups. A *p* value of less than 0.05 was considered statistically significant for comparisons between tumor locations, and a *p* value of less than 0.05/4 = 0.0125 using Bonferroni correction for comparisons between CT scanners and between reconstruction methods. Moreover, decision curve analysis (DCA) was performed to assess the clinical utility of using our method in predicting the presence or absence of pancreatic cancer [16, 17]. Additionally, cases missed by the software or radiologists were thoroughly evaluated. To assess the accuracy of tumor localization in detecting PDAC, the software’s results were compared with findings from EUS, which has been reported to be superior to multidetector CT for PDAC detection in a previous prospective observational study [18]. The location of the pancreatic cancer detected in the software was defined as the location, where the heat map responds to pancreatic cancer-like structures observable on the image, and otherwise as the location, where the D/P ratio value increases sharply from the pancreatic head side to the pancreatic tail side. The location of the pancreatic cancer detected in the software was compared to the location of the pancreatic cancer detected with EUS. Accuracy analysis of the present software was performed by one cholangio-pancreatic endoscopist (S.H. with 27 years of experience). All statistical analyses were performed using the R software (R Foundation, Vienna, Austria). Statistical significance was determined using a *p* value < 0.05.

## Results

### Data set characteristics

The demographic and clinical characteristics of the patients are summarized in Table 1. The mean tumor size measured by the axial image of CT was 16 mm (range: 7–20 mm). The pancreatic head was the most common tumor location (49/100 cases, including 6 cases of the uncinate process), and most tumors (91/100 cases) were stage IA (UICC, for International Cancer Control 8th edition).

**Table 1:** Characteristics of patients and tumors

	Pancreatic adenocarcinoma (*n* = 100)	Control (*n* = 104)
Age (y)		
Median (range)	72 (41–89)	66.5 (36–83)
Sex		
Male/Female	54/46	64/40
Tumor size (mm)		
Median (range)	16 (7–20)	
Tumor location		
Head (uncinate process) Head (the other)/body/tail	6/43/33/18	
Stage (UICC 8th edition)		
0	0	
IA	91	
IB	0	
IIA	0	
IIB	5	
III	0	
IV	4	

### Performance of each model

**Fig. 3:**
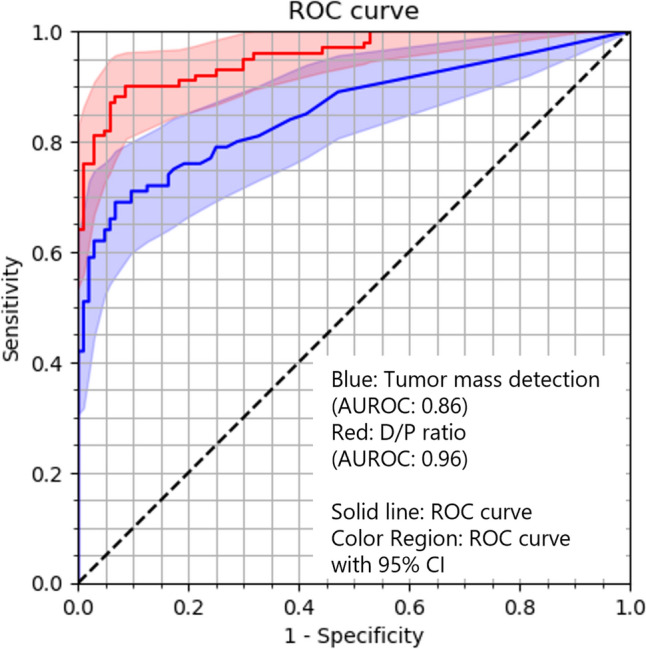
Receiver operating characteristic (ROC) curve analyses of tumor mass detection and D/P ratio

**Fig. 4:**
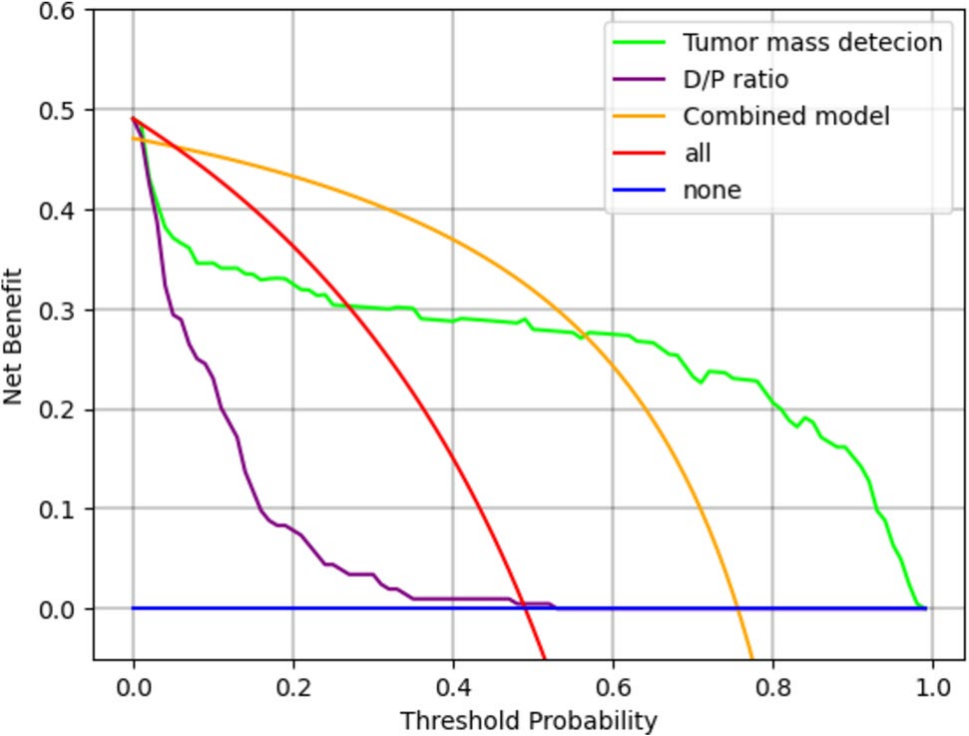
Decision curve analysis (DCA) of tumor mass detection, D/P ratio, and combined model

Threshold values were 0.051 for tumor mass detection and 0.019 for the D/P ratio. The overall sensitivity, specificity, PPV, and NPV of tumor mass detection were 77.0% (CI 68.8–85.2%), 76.0% (CI 67.7–84.2%), 75.5% (CI 68.9–82.1%), and 77.5% (CI 70.9–84.0%), respectively; those of the D/P ratio detection were 87.0% (CI 80.4–93.6%), 94.2% (CI 89.8–98.7%), 93.5% (CI 88.8–98.3%), and 88.3% (CI 83.0–93.6%), respectively; and those of the combination of tumor mass detection and the D/P ratio detection were 96.0% (CI 92.2–99.8%), 70.2% (CI 61.4–79.0%), 76.0% (CI 70.1–81.1%), and 94.8% (CI 90.0–99.6%), respectively. The results are summarized in Table 2 and Fig. 3. Results classified by CT scanner and reconstruction, and tumor location are shown in Tables S2, S3, and 3, respectively. Of the 100 PDAC cases, 6 were classified as pancreatic head cancer (uncinate process), 43 as pancreatic head cancer (the other), 33 as pancreatic body cancer, and 18 as pancreatic tail cancer. Fisher’s exact test revealed that there were no significant differences in the sensitivity between the pancreatic tumor locations. There were no significant differences in the sensitivity and specificity between CT scanners. There were no significant differences in the sensitivity, specificity, and NPV between reconstruction. The results of DCA are showed in Fig. 4.

**Table 2:** Sensitivity and specificity of heat map, D/P ratio, and combination of heat map and D/P ratio

	Heat map	D/P ratio	Combination
Sensitivity	77.0%	87.0%	96.0%
Specificity	76.0%	94.2%	70.2%
PPV	75.5%	93.5%	75.6%
NPV	77.5%	88.3%	94.8%

*PPV* positive predictive value, *NPV* negative predictive value

### Comparison of sensitivity with radiologist interpretation

The exact McNemar test revealed that there was no significant difference between the sensitivity of our software and that of the original radiologist’s report (software, 96.0%; radiologist’s report, 96.0%; *p* = 1). The software missed four PDACs, and four were also missed by the radiologist. The cases missed by the software and the radiologists were completely different Table 3.

**Table 3:** Performance classified by pancreatic tumor location

Pancreatic tumor location	Number	Tumor mass detection	D/P ratio	Combined model
		Sensitivity (95% CI)	Sensitivity (95% CI)	Sensitivity (95% CI)
Head (uncinate process)	PDAC:6	83.3% (53.5–100%)	66.7% (28.9–100%)	83.3% (53.5–100%)
Head (the other)	PDAC:43	81.4% (69.8–93.0%)	81.4% (69.8–93.0%)	95.3% (89.1–100%)
Body	PDAC:33	78.8% (64.8–92.7%)	93.9% (85.8–100%)	97.0% (91.1–100%)
Tail	PDAC:18	61.1% (38.6–83.6%)	94.4% (83.9–100%)	100% (100–100%)
*p* value		0.360	0.119	0.362
Total	PDAC:100			

*PDAC* pancreatic ductal adenocarcinoma

### Accuracy in detecting PDAC of software

The coincident ratio of tumor localization between the findings of the present software and those of EUS was 96.0%. In four cases, the location of the tumor detected by the present software differed from that detected by EUS.

## Discussion

**Fig. 5:**
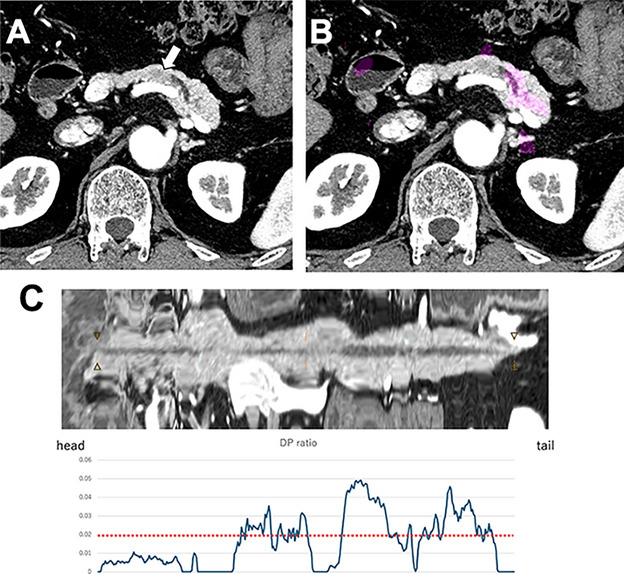
Images from a 61-year-old male with pathologically proven pancreatic ductal adenocarcinoma.** a** Pancreatic phase of contrast-enhanced CT images revealed an 18 mm pancreatic ductal adenocarcinoma on the pancreatic body (white arrow);** b** tumor probability map (heat map) image could not shade the tumor; **c** D/P ratio elevated above the threshold (red dotted line), thereby suggesting the provability of pancreatic ductal adenocarcinoma. Finally, this case was judged as pancreatic ductal adenocarcinoma

Improving the detectability of small PDAC remains an unmet clinical need. Our proposed software accurately demonstrated high diagnostic performance while accurately detecting small PDAC with a sensitivity of 96.0% and achieving a concordance rate of 96.0% with EUS findings. Furthermore, our results suggest that incorporating indirect indicator evaluation into automatic tumor mass detection could enhance the accuracy of small PDAC detection (Fig. 5).

Indirect indicators such as MPD dilatation play a vital role in identifying small PDAC, and their clinical importance has been previously reported [18, 19]. A retrospective observational study demonstrated that 88% of isoattenuating small PDAC cases present are indirect indicators [20]. Another multicenter retrospective observational study reported that MPD dilatation and focal fatty changes in the pancreatic parenchyma were observed in 79.6% and 41.8% of patients with small PDAC, respectively [10]. Previous studies on deep learning-based automated detection of PDAC have mainly focused on tumor mass detection, which may have led to the omission of small PDAC cases, particularly isoattenuating tumors. Deep learning-based automated detection tools have been reported to achieve diagnostic performance comparable to that of experienced radiologists for detecting small PDACs [6]. However, their sensitivity remains suboptimal, and ranges from 74.7 to 96.0% [5–7, 9]. Identifying indirect indicators often leads to further examinations, such as EUS, which has been reported to be more sensitive than CT [21]. In this study, we integrated automatic tumor mass detection with indirect indicator evaluation, thereby mirroring the approach radiologists employ in daily clinical practice. Our findings suggest that combining automatic tumor mass detection with indirect indicator evaluation has the potential to improve diagnostic accuracy.

Our results suggest that integrating indirect indicators improved sensitivity but led to a slight reduction in specificity. This reduction in specificity may be attributed to the fact that cases with positive results in either automatic tumor mass detection or the D/P ratio were classified as PDAC. The results of the DCA for developed model showed that combined model had a higher net benefit than tumor mass detection and the D/P ratio in the range of threshold probabilities below 0.5. In situations where the benefit of finding true positive patients outweighs the harm of intervening in false positive controls, such as pancreatic cancer screening, the combined model may be more beneficial than tumor mass detection and the D/P ratio as it enables the identification of more true positive patients.

Moreover, in this study, the cases missed by our software differed from those missed by the radiologists. In three of the four cases missed by the software, the tumor was located in the uncinate process of the pancreas, and no indirect indicators were present. In all three of these cases, the software failed to detect the tumor mass. Our software also failed to detect the tumor mass or indirect indicators in the remaining case. In this case, neither the radiologist nor the software could detect the tumor mass. However, the MPD was slightly dilated compared to previous studies (3 mm in diameter, not considered significant), thereby leading the radiologist to suspect PDAC. Conversely, the four cases missed by the radiologists were identified to be PDAC by our software. However, the lesions detected by our software in these four cases were not PDAC. The structures misdiagnosed as PDAC included inflammation in two cases and common bile duct and pancreatic cysts in one case each, thereby highlighting the need for refined algorithms to reduce such false positives. PDACs located in the uncinate process of the pancreas may be misdiagnosed by our software as they often lack indirect indicators. Additionally, the software cannot evaluate changes in CT images over time, which may result in the oversight of subtle changes in indirect indicators.

The present study also has some limitations. First, it was a retrospective study conducted at a single institution. Since our institution is a tertiary referral hospital with a high volume of PDAC patients, the difference in sensitivity between the software and radiologists’ interpretations may not be generalizable to other settings. Second, we assessed only the pancreatic phase. To accurately assess the potential usefulness of our software, future studies should evaluate its performance across additional contrast phases, as dynamic contrast-enhanced CT is not always available. Third, the software carries a potential risk of overdiagnosis as indirect findings do not always indicate the presence of PDAC. This risk could be mitigated by incorporating the evaluation of image changes over time, which is an important area for future research. Fourth, we set thresholds for 80% sensitivity and 100% specificity in the threshold-setting cases. Thresholds should ideally be determined using data sets that encompass a wider range of variation. Finally, we included 100 PDAC patients and 104 controls as the testing data set, which was artificial PDAC/control ratio. In clinical practice, PPV and NPV may differ from those observed in this study.

In conclusion, the software evaluated in this study demonstrated that combining automated tumor mass detection with indirect indicator evaluation may improve PDAC detection.

## Supplementary Information

Below is the link to the electronic supplementary material.

Supplementary file1 (DOCX 164 KB)

## References

[1] Rahib L, Smith BD, Aizenberg R, Rosenzweig AB, Fleshman JM, Matrisian LM. Projecting cancer incidence and deaths to 2030: the unexpected burden of thyroid, liver, and pancreas cancers in the United States. Cancer Res. 2014;74:2913–21. 10.1158/0008-5472.can-14-0155. 24840647 10.1158/0008-5472.CAN-14-0155

[2] Chari ST. Detecting early pancreatic cancer: problems and prospects. Semin Oncol. 2007;34:284–94. 10.1053/j.seminoncol.2007.05.005. 17674956 10.1053/j.seminoncol.2007.05.005 PMC2680914

[3] Chu LC, Park S, Kawamoto S, Park S, Kawamoto S, Fouladi DF, et al. Utility of CT radiomics features in differentiation of pancreatic ductal adenocarcinoma from normal pancreatic tissue. AJR Am J Roentgenol. 2019;213:349–57. 10.2214/ajr.18.20901. 31012758 10.2214/AJR.18.20901

[4] Kang JD, Clarke SE, Costa AF. Factors associated with missed and misinterpreted cases of pancreatic ductal adenocarcinoma. Eur Radiol. 2021;31:2422–32. 32997176 10.1007/s00330-020-07307-5

[5] Cao K, Xia Y, Yao J, Han X, Lambert L, Zhang T, et al. Large-scale pancreatic cancer detection via non-contrast CT and deep learning. Nat Med. 2023;29:3033–43. 10.1038/s41591-023-02640-w. 37985692 10.1038/s41591-023-02640-w PMC10719100

[6] Chen PT, Wu T, Wang P, Chang D, Liu KL, Wu MS, et al. Pancreatic cancer detection on ct scans with deep learning: a nationwide population-based study. Radiol. 2023;306:172–82. 10.1148/radiol.220152. 10.1148/radiol.220152 36098642

[7] Korfiatis P, Suman G, Patnam NG, Trivedi KH, Karbhari A, Mukherjee S, et al. Automated artificial intelligence model trained on a large data set can detect pancreas cancer on diagnostic computed tomography scans as well as visually occult preinvasive cancer on prediagnostic computed Tomography Scans. Gastroenterol. 2023;165(1533–1546): e1534. 10.1053/j.gastro.2023.08.034. 10.1053/j.gastro.2023.08.034 PMC10843414 37657758

[8] Ma H, Liu ZX, Zhang JJ, Wu FT, Xu CF, Shen Z, et al. Construction of a convolutional neural network classifier developed by computed tomography images for pancreatic cancer diagnosis. World J Gastroenterol. 2020;26:5156–68. 10.3748/wjg.v26.i34.5156. 32982116 10.3748/wjg.v26.i34.5156 PMC7495037

[9] Liu KL, Wu T, Chen PT, Tsai YM, Roth H, Wu MS, et al. Deep learning to distinguish pancreatic cancer tissue from non-cancerous pancreatic tissue: a retrospective study with cross-racial external validation. Lancet Digit Health. 2020;2:e303–13. 10.1016/s2589-7500(20)30078-9. 33328124 10.1016/S2589-7500(20)30078-9

[10] Kanno A, Masamune A, Hanada K, Maguchi H, Shimizu Y, Ueki T, et al. Multicenter study of early pancreatic cancer in Japan. Pancreatol. 2018;18:61–7. 10.1016/j.pan.2017.11.007 29170051

[11] Abi Nader C, Vetil R, Wood LK, Rohe MM, Bône A, Karteszi H, et al. Automatic detection of pancreatic lesions and main pancreatic duct dilatation on portal venous CT scans using deep learning. Invest Radiol. 2023;58(11):791–8. 10.1097/RLI.0000000000000992. 37289274 10.1097/RLI.0000000000000992

[12] Hattori C, Furukawa D, Yamazaki F, Fujisawa Y, Sakaguchi T. Centerline detection and estimation of pancreatic duct from abdominal CT images. SPIE; 2022.

[13] Ambo S, Hirano R, Hattori C. Automatic detection of main pancreatic duct dilation and pancreatic parenchymal atrophy based on a shape feature in abdominal contrast-enhanced CT images. J Med Imaging (Bellingham). 2025;12: 014504. 10.1117/1.jmi.12.1.014504. 39895855 10.1117/1.JMI.12.1.014504 PMC11782102

[14] Roth HR, Farag A, Turkbey EB, Le Lu JL, Summers RM. Data from pancreas-CT. The Cancer Imaging Archive Public Access. 10.7937/K9/TCIA.2016.tNB1kqBU. published 2016. Accessed 24 Nov 2021.

[15] ArXivLabs. Medical segmentation decathlon. http://medicaldecathlon.com. published June 2021. Accessed 24 Nov 2021

[16] Zhang Z, Rousson V, Lee WC, Ferdynus C, Chen M, Qian X, et al. Decision curve analysis: a technical note. Annals Trans Med. 2018;6(15):308–18. 10.21037/atm.2018.07.02. 10.21037/atm.2018.07.02 PMC6123195 30211196

[17] Vickers AJ, Elkin EB. Decision curve analysis: a novel method for evaluating prediction models. Med Decis Making. 2006;26(6):565–74. 10.1177/0272989X06295361. 17099194 10.1177/0272989X06295361 PMC2577036

[18] DeWitt J, Devereaux B, Chriswell M, McGreevy K, Howard T, Imperiale TF, et al. Comparison of endoscopic ultrasonography and multidetector computed tomography for detecting and staging pancreatic cancer. Ann Intern Med. 2004;141:753–63. 10.7326/0003-4819-141-10-200411160-00006. 15545675 10.7326/0003-4819-141-10-200411160-00006

[19] Prokesch RW, Chow LC, Beaulieu CF, Bammer R, Jeffrey RB Jr. Isoattenuating pancreatic adenocarcinoma at multi-detector row CT: secondary signs. Radiol. 2002;224:764–8. 10.1148/radiol.2243011284. 10.1148/radiol.2243011284 12202711

[20] Yoon SH, Lee JM, Cho JY, Lee KB, Kim JE, Moon SK, et al. Small (</= 20 mm) pancreatic adenocarcinomas: analysis of enhancement patterns and secondary signs with multiphasic multidetector CT. Radiol. 2011;259:442–52. 10.1148/radiol.11101133. 10.1148/radiol.11101133 21406627

[21] Dewitt J, Devereaux BM, Lehman GA, Sherman S, Imperiale TF. Comparison of endoscopic ultrasound and computed tomography for the preoperative evaluation of pancreatic cancer: a systematic review. Clin Gastroenterol Hepatol. 2006;4(717–725):664. 10.1016/j.cgh.2006.02.020. 16675307 10.1016/j.cgh.2006.02.020

