# Supplementary material for: Deep learning-based automatic detection of pancreatic ductal adenocarcinoma ≤ 2 cm with high-resolution computed tomography: impact of the combination of tumor mass detection and indirect indicator evaluation
Source: Jpn J Radiol. 2025 Jul 18;43(11):1870–7. doi: 10.1007/s11604-025-01836-z (PMC12575530; doi:10.1007/s11604-025-01836-z)

**Table S1.** Detailing CNN architecture and learning conditions

|  | Conditions |
| --- | --- |
| CNN architecture | Residual squeeze and excitation U-Net |
| Loss function | Mean squared error |
| Optimizer | Adam |
| Epochs | 500 (Before fine tuning)  250 (After fine tuning) |
| Batch size | 4 |
| Learning rate | 1e-4 |
| Augmentation | ±10° z-axis rotation  ±7% x-axis shift  ±7% y-axis shift  ±7% z-axis shift  90% – 110% scaling  X-axis flip, Y-axis flip, Z-axis flip  Window Level: 50 – 70 HU  Window width: 330 – 370 HU |

**Table S2**. Performance classified by CT scanner

| CT scanner | Number | Tumor mass detection | | | | D/P ratio | | | | Combined model | | | |
| --- | --- | --- | --- | --- | --- | --- | --- | --- | --- | --- | --- | --- | --- |
|  |  | Sen.  (95% CI) | Spe.  (95% CI) | PPV  (95% CI) | NPV  (95% CI) | Sen.  (95% CI) | Spe.  (95% CI) | PPV  (95% CI) | NPV  (95% CI) | Sen.  (95% CI) | Spe.  (95% CI) | PPV  (95% CI) | NPV  (95% CI) |
| Aquilion ONE | PDAC:5, control:32 | 80.0%  (44.9-100%) | 75.0%  (60.0-90.0%) | 33.3%  (16.8-49.8%) | 96.0%  (89.2-100%) | 100%  (100-100%) | 100%  (100-100%) | 100%  (100-100%) | 100%  (100-100%) | 100%  (100-100%) | 75.0%  (60.0%-90.0%) | 38.5%  (24.3-52.7%) | 100%  (100%-100%) |
| Aquilion PRIME | PDAC:18, control:71 | 66.7%  (44.9-88.4%) | 76.1%  (66.1-86.0%) | 41.4%  (28.6-54.2%) | 90.0%  (84.0-96.0%) | 83.3%  (66.1-100%) | 91.5%  (85.1-98.0%) | 71.4%  (55.2-87.6%) | 95.6%  (91.2-100%) | 88.9%  (74.3-100%) | 67.6%  (56.7-78.5%) | 71.4%  (32.0-50.1%) | 96.0%  (90.9-100%) |
| Aquilion Precision | PDAC:77, control:1 | 79.2%  (70.2-88.3%) | 100%  (100-100%) | 100%  (100-100%) | 5.9%  (3.5-8.3%) | 87.0%  (79.5-94.5%) | 100%  (100-100%) | 100%  (100-100%) | 9.1%  (4.3-13.9%) | 97.4%  (93.8-100%) | 100%  (100-100%) | 100%  (100-100%) | 33.3%  (2.9-63.7%) |
| P value |  | 0.499 | 1 | <0.001 | <0.001 | 0.856 | 0.221 | <0.001 | <0.001 | 0.319 | 0.646 | <0.001 | <0.001 |
| Total | PDAC:100, control:104 |  |  |  |  |  |  |  |  |  |  |  |  |

Sen., sensitivity; Spe., specificity; PPV, positive predictive value; NPV, negative predictive value; PDAC, pancreatic ductal adenocarcinoma.

**Table S3**. Performance classified by reconstruction

| Reconstruction | Number | Tumor mass detection | | | | D/P ratio | | | | Combined model | | | |
| --- | --- | --- | --- | --- | --- | --- | --- | --- | --- | --- | --- | --- | --- |
|  |  | Sen.  (95% CI) | Spe.  (95% CI) | PPV  (95% CI) | NPV  (95% CI) | Sen.  (95% CI) | Spe.  (95% CI) | PPV  (95% CI) | NPV  (95% CI) | Sen.  (95% CI) | Spe.  (95% CI) | PPV  (95% CI) | NPV  (95% CI) |
| AIDR3D | PDAC:32, control:72 | 71.9%  (56.3-87.5%) | 76.3%  (66.6-86.2%) | 57.5%  (46.0-69.0%) | 85.9%  (79.1-92.8%) | 84.4%  (71.8-97.0%) | 91.7%  (85.3-98.1%) | 81.8%  (70.2-93.4%) | 93%  (87.7-98.2%) | 90.6%  (80.5-100%) | 68.1%  (57.3-78.8%) | 55.8%  (47.0-64.5%) | 94.2%  (88.3-100%) |
| AiCE | PDAC:68, control:32 | 79.4%  (69.8-89.0%) | 75.0%  (60.0-90.0%) | 87.1%  (80.2-94.0%) | 63.2%  (51.3-75.0%) | 88.2%  (80.6-95.9%) | 100%  (100-100%) | 100%  (100-100%) | 80.0%  (69.9-90.4%) | 98.5%  (95.7-100%) | 75.0%  (60.0-90.0%) | 89.3%  (83.6-95.1%) | 96.0%  (88.5-100%) |
| P value |  | 0.45 | 1 | <0.001 | 0.013 | 0.751 | 0.174 | <0.001 | 0.063 | 0.095 | 0.643 | <0.001 | 1 |
| Total | PDAC:100, control:104 |  |  |  |  |  |  |  |  |  |  |  |  |

Sen., sensitivity; Spe., specificity; PPV, positive predictive value; NPV, negative predictive value; PDAC, pancreatic ductal adenocarcinoma.


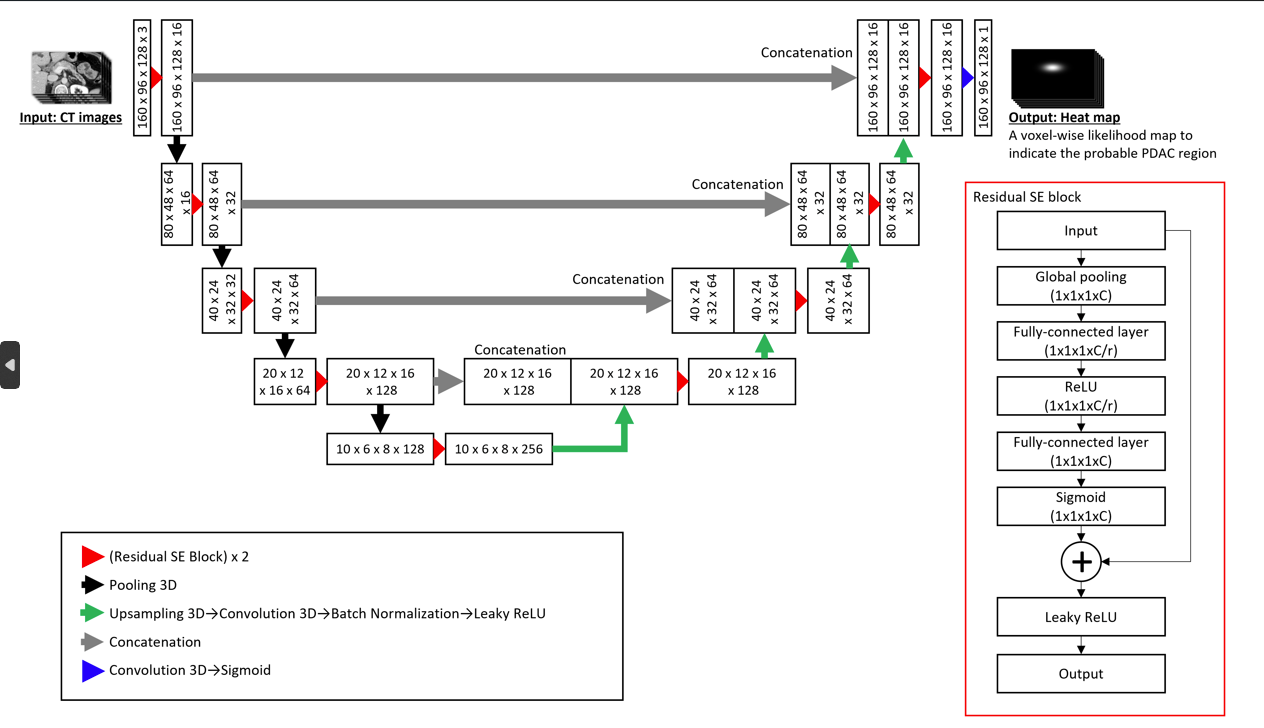

Supplement: Supplementary file 1 — Supplementary file1 (DOCX 164 KB) [file 11604_2025_1836_MOESM1_ESM.docx]
